# Stability and predictive value of anti-JCV antibody index in multiple sclerosis: A 6-year longitudinal study

**DOI:** 10.1371/journal.pone.0174005

**Published:** 2017-03-20

**Authors:** Harald Hegen, Michael Auer, Gabriel Bsteh, Franziska Di Pauli, Tatiana Plavina, Janette Walde, Florian Deisenhammer, Thomas Berger

**Affiliations:** 1 Department of Neurology, Medical University of Innsbruck, Innsbruck, Austria; 2 Biogen, Cambridge, Massachusetts, United States of America; 3 Department of Statistics, Faculty of Economics and Statistics, University of Innsbruck, Innsbruck, Austria; Charite Universitatsmedizin Berlin, GERMANY

## Abstract

**Background:**

Risk of natalizumab-related progressive multifocal leukoencephalopathy is associated with the presence of anti-JC-virus (JCV) antibodies.

**Objective:**

To investigate the longitudinal evolution of anti-JCV antibody index and to determine the predictive value of baseline anti-JCV antibody index for long-term stability of anti-JCV antibody status.

**Methods:**

MS patients from the MS centre of Medical University of Innsbruck, who had serum sampling for a time period of 4–6 years at intervals of 6±3 months, were included in this retrospective, longitudinal study. Anti-JCV antibody serological status and index were determined by 2-step second-generation anti-JCV antibody assay.

**Results:**

154 patients were included in this study. Median follow-up time was 63.7 months, with median 11 samples available per patient. At baseline, 111 (72.1%) patients were anti-JCV antibody positive. Baseline anti-JCV antibody index significantly correlated with age (R = 0.22, p = 0.005); there was no difference with respect to sex, disease duration or previously used disease-modifying treatment. During follow-up anti-JCV antibody status changed from negative to positive or vice versa in 17% of patients. In seronegative patients at baseline, baseline anti-JCV antibody index was significantly lower in those remaining seronegative at follow-up compared to those converting to seropositivity (median 0.16 vs. 0.24, p = 0.002). In seropositive patients at baseline, index was higher in those remaining seropositive compared to those reverting to seronegativity (2.6 vs. 0.45, p<10^−7^). Baseline anti-JCV antibody index >0.90 predicted stable positive serostatus (sensitivity 88.7%, specificity 96.5%) and <0.20 stable negative serostatus (sensitivity 61.3%, specificity 97.6%).

**Conclusions:**

Anti-JCV antibody index remained relatively stable over 6-year follow-up with annual serostatus change of ~3%. Baseline anti-JCV antibody index predicted stable negative and stable positive JCV serostatus.

## Introduction

Natalizumab is an effective disease-modifying therapy (DMT) for patients with relapsing multiple sclerosis (MS) [1]. However, natalizumab treatment is associated with the risk of progressive multifocal leukoencephalopathy (PML), an opportunistic infection of the brain caused by John Cunningham virus (JCV) [2]. Several factors such as prior use of immunosuppressants, duration of natalizumab treatment and especially presence of serum anti-JCV antibodies determine PML risk [3]. Anti-JCV antibodies occur in 50–70% of MS patients [4–7] and, recently, anti-JCV antibody index has been reported to correlate with PML risk in seropositive patients [8]. Although it is reported that seroconversion occurs in approximately 2–15% of patients per year [5, 8–14], there is insufficient evidence on long-term dynamics of anti-JCV antibody index. We performed this study to better understand the evolution of anti-JCV antibody index and to optimize reliable benefit-risk evaluations prior and during natalizumab treatment.

We aimed to investigate the longitudinal evolution of anti-JCV antibody index in MS patients, to evaluate the impact of age, sex, disease duration, disease course and DMT, and to determine the predictive value of baseline anti-JCV antibody index for long-term stability of anti-JCV antibody status.

## Materials and methods

### Patients and samples

Patients with MS [15] or clinically isolated syndrome [16], who were seen at the MS centre of Medical University of Innsbruck between September 2004 and March 2010, with and without current DMT, and who had routine blood sampling for a time period of 4–6 years at intervals of 6±3 months (up to two samples were allowed to be missed in between), were included in this retrospective, longitudinal study. All blood sampling had been performed at the treating physicians’ discretion during patients’ routine clinical visits. The disease course of MS patients was classified based on Lublin and Reingold [17]. Patients with prior intravenous immunoglobulin therapy were excluded to avoid potential influence on anti-JCV antibodies [18]. Blood samples were collected by peripheral venous puncture. Serum was isolated from blood by centrifugation, after the blood samples were allowed to clot for ≥30 minutes. All samples were stored at the Neuroimmunology Laboratoy of Medical University of Innsbruck at -20°C until analysis, which was centrally performed at Unilabs (Copenhagen, Denmark) at one time point in 2015 and only for the purpose of this study.

### Anti-JCV antibody assay

Anti-JCV antibody serological status and index were determined by a two-step enzyme-linked immunosorbent assay (STRATIFY JCV DxSelect; Focus Diagnostics, Cypress; CA, USA). For detailed assay description see [8, 19].

An anti-JCV antibody index >0.40 denoted anti-JCV antibody positivity and index <0.20 denoted anti-JCV antibody negativity. For samples with an index ≥0.20 but ≤0.40 (intermediate response) further evaluation in the confirmation test (second step) was required. In the confirmation test, patient sample is preinhibited with the coating antigen in solution and, then, the preinhibited and noninhibited aliquots of patient serum are tested. The results of the confirmation assay are reported as percentage inhibition, calculated as 100 × (1- (optical density of preinhibited/ noninhibited sample)). Samples were scored eventually positive when inhibition was >45% [8, 19].

### Definition of seroconversion and seroreversion

Seroconversion was defined as positive anti-JCV antibody result at least once during follow-up, if baseline serostatus was negative. Seroreversion was defined as negative anti-JCV antibody testing at least once during the observation period in case of baseline positive serostatus.

### Statistic analysis

We performed statistical analysis using SPSS 23.0 (SPSS Inc, Chicago, IL, USA). Distribution of data was assessed by Kolmogorov-Smirnov test and data were displayed as mean ± standard deviation or as median and interquartile range (IQR). Change of anti-JCV antibody index over time was assessed by Friedman test. For group comparisons of anti-JCV antibody index (at a certain time point), e.g. between males and females or different types of disease course, Mann-Whitney U or Kruskal-Wallis Test were applied. Qualitative variables, such as anti-JCV antibody frequency, were compared between groups using chi-square-test or Fisher’s exact test. Spearman coefficient was used for correlation analysis. We assessed sensitivities, specificities, positive and negative predictive values of baseline anti-JCV antibody index for stability of JCV serostatus by receiver operating characteristic (ROC) analysis. Multinomial logistic regression analysis was employed to investigate predictors for stable or non-stable behaviour of JCV serostatus. The power of the predictors was calculated as risk ratio of the percentage correctly predicted cases (hit ratio; HR) and the HR by chance (the empirical distribution of patient groups). Two-tailed p-values <0.05 were considered as statistically significant.

### Ethics

The study was approved by the ethics committee of Medical University of Innsbruck (approval number AN2014-0347 344/4.8). Written informed consent was obtained from all patients.

## Results

A total of 154 patients were included into this study. Clinical and baseline demographic data are shown in Table 1. Median follow-up time was 63.7 months (IQR 55.1–69.6) with median of 11 (range 7–13) longitudinally collected samples available per patient.

**Table 1:** Clinical and baseline demographic data.

Number of patients	154
Age (years), mean±SD (range)	37.5±10.7 (18–70)
Sex (female), n (%)	109 (70.8)
Disease course, n (%)	
CIS	5 (3.2)
RRMS	115 (74.7)
SPMS with relapses	16 (10.4)
SPMS	11 (7.1)
PPMS	7 (4.5)
Disease duration (years), median (IQR)	5.9 (2.5–10.7)
Previous use of DMT, n (%)	
Immunosuppressants	
Azathioprine	15 (9.7)
Cyclophosphamide/ mitoxantrone	6 (3.9)
Natalizumab	2 (1.3)
Interferon-β	88 (57.1)
Glatiramer acetate	16 (10.4)
Fingolimod	0 (0)
No previous DMT	48 (31.2)
Treatment duration of prior DMT (months), median (IQR)	
Immunosuppressants	
Azathioprine	45.0 (17.0–83.2)
Cyclophosphamide/ mitoxantrone	10.4 (5.1–17.7)
Natalizumab	5.3*
Interferon-β	23.0 (9.1–50.6)
Glatiramer acetate	13.9 (3.5–45.0)
Use of DMT during observation period, n (%)	
Immunosuppressants	
Azathioprine	7 (4.5)
Cyclophosphamide	1 (0.6)
Natalizumab	31 (20.1)
Interferon-β	99 (64.3)
Glatiramer acetate	22 (14.3)
Fingolimod	4 (2.6)
No DMT	27 (17.5)
Treatment duration during observation period (months), median (IQR)	
Immunosuppressants	
Azathioprine	37.0 (25.6–61.4)
Cyclophosphamide	18.2*
Natalizumab	37.5 (24.2–57.1)
Interferon-β	45.5 (26.2–60.8)
Glatiramer acetate	36.5 (17.4–54.6)
Fingolimod	25.6*

* IQR not given due to low number of patients.

CIS, clinically isolated syndrome; DMT, disease-modifying treatment; IQR, interquartile range; n, number; PPMS, primary progressive multiple sclerosis; RRMS, relapsing-remitting multiple sclerosis; SD, standard deviation; SPMS, secondary progressive multiple sclerosis

### Anti-JCV serological status and antibody index at baseline

**Fig 1:**
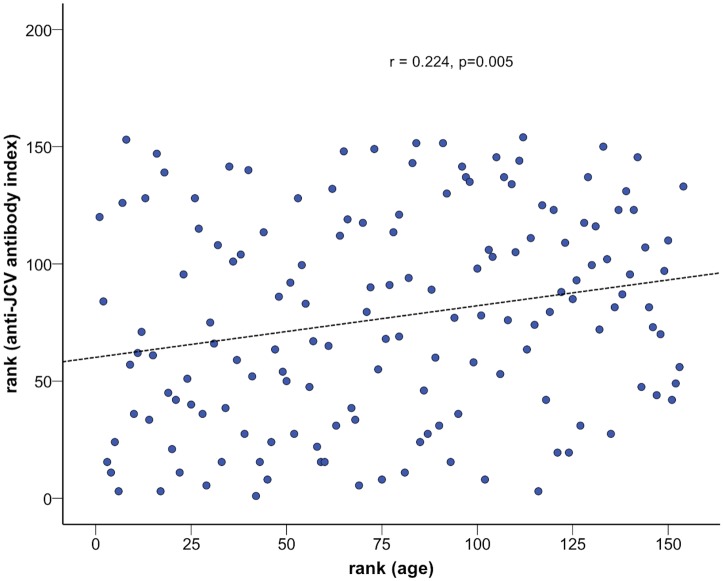
Spearman correlation between anti-JCV antibody index and patients’ age. The correlation between the rank of patient's age and the rank of anti-JVC antibody index is visualized, i.e. the Spearman correlation coefficient with its significance.

Of the 154 patients, 111 (72.1%) were anti-JCV antibody positive at baseline with a median anti-JCV antibody index of 2.3 (IQR 1.1–3.1). Within the anti-JCV antibody negative patient group median index was 0.19 (IQR 0.15–0.23). Anti-JCV antibody index statistically significantly correlated with patients’ age (R = 0.22, p = 0.005; Fig 1), did not differ between males and females or the different types of disease course, and there was no correlation with disease duration. Anti-JCV antibody index did not significantly differ between patients with and without any previous DMT (i.e. any previous immunomodulatory or immunosuppressive therapy).

### Evolution of anti-JCV serological status and antibody index during 6 years follow-up

**Fig 2:**
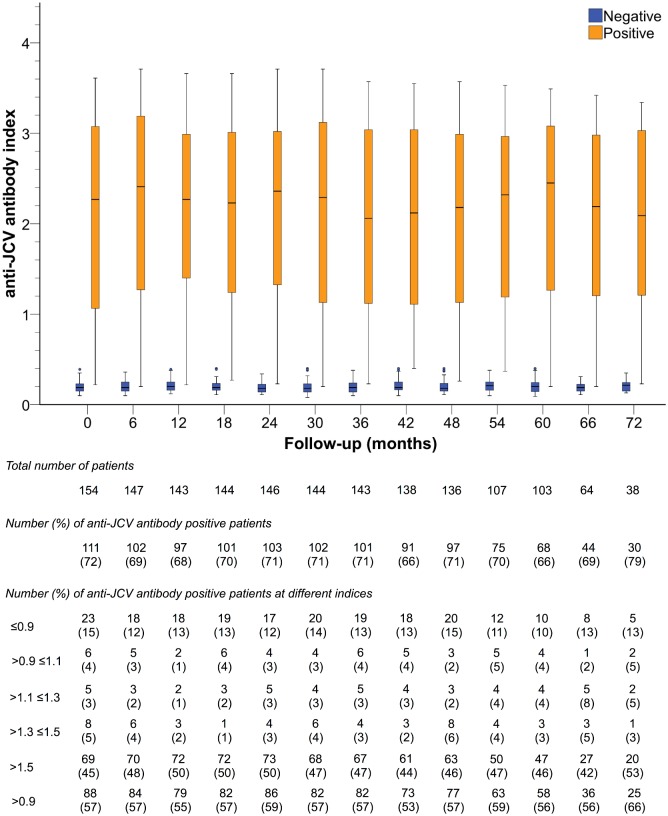
Evolution of anti-JCV antibody index. Anti-JCV antibody indices are displayed in seronegative and seropositive patients at the different time points during follow-up. At each visit, the total number of patients, the number of anti-JCV antibody positive patients and their distribution among different index categories are shown. JCV, John Cunningham virus.

Overall, anti-JCV antibody index did not significantly vary within the observation period (Fig 2). Until the last follow-up, 128 (83.1%) patients did not change serological status. There was no difference between patients who changed initial serostatus and those who did not regarding to age, sex, disease duration, disease course or prior/ current use of DMT (i.e. any prior/ current immunomodulatory or immunosuppressive therapy).

### Seroconversion

**Fig 3:**
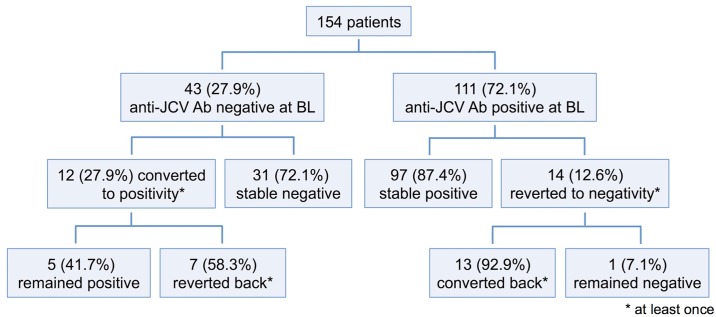
Longitudinal change of anti-JCV serostatus. Frequency of conversion and reversion of anti-JCV serostatus from baseline throughout follow-up.

**Fig 4:**
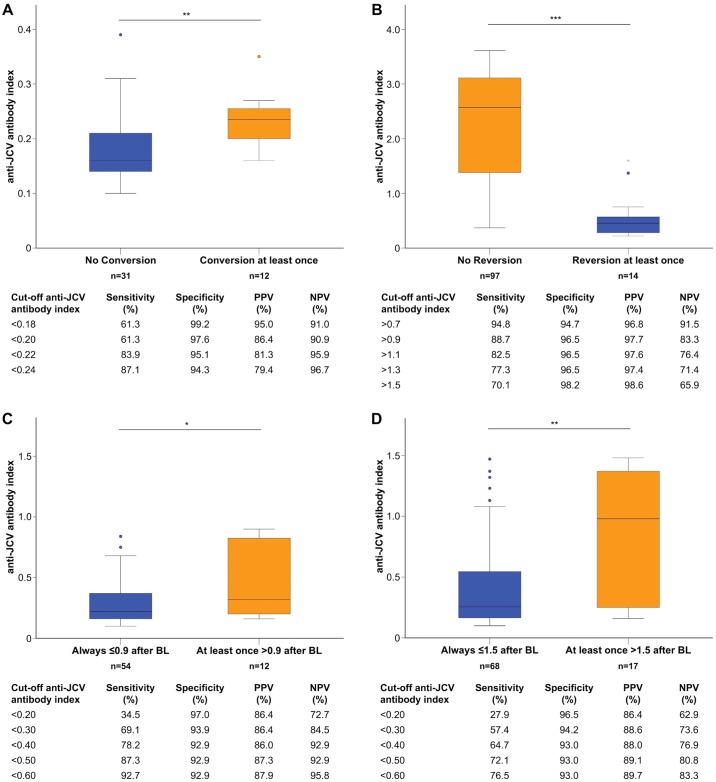
Predictive value of baseline anti-JCV antibody index. (A) Anti-JCV antibody index in seronegative patients at baseline according to seroconversion status at follow-up. Predictive value of different anti-JCV antibody index levels at baseline for prediction of stable seronegative status at follow-up (AUC: 0.986, p<10^−23^). (B) Anti-JCV antibody index in seropositive patients at baseline according to seroreversion status at follow-up. Predictive value of different anti-JCV antibody index levels at baseline for prediction of stable seropositive status at follow-up (AUC: 0.976, p<10^−15^). (C) Anti-JCV antibody index in patients with baseline values ≤0.9 according to the change to a higher index category (>0.9) at follow-up. Predictive value of different anti-JCV antibody index levels at baseline for prediction of stable JCV index ≤0.9 at follow-up (AUC: 0.962, p<10^−20^). (D) Anti-JCV antibody index in patients with baseline values ≤1.5 according to the change to a higher index category (>1.5) at follow-up. Predictive value of different anti-JCV antibody index levels at baseline for prediction of stable JCV index ≤1.5 at follow-up (AUC: 0.948, p<10^−20^). Ab, antibody; AUC, area under the curve; BL, baseline; JCV, John Cunningham virus; NPV, negative predictive value; PPV, positive predictive value; ROC, receiver operating characteristic; **, *** indicate statistical significance at a P-value <0.01 and <0.001, respectively.

**Fig 5:**
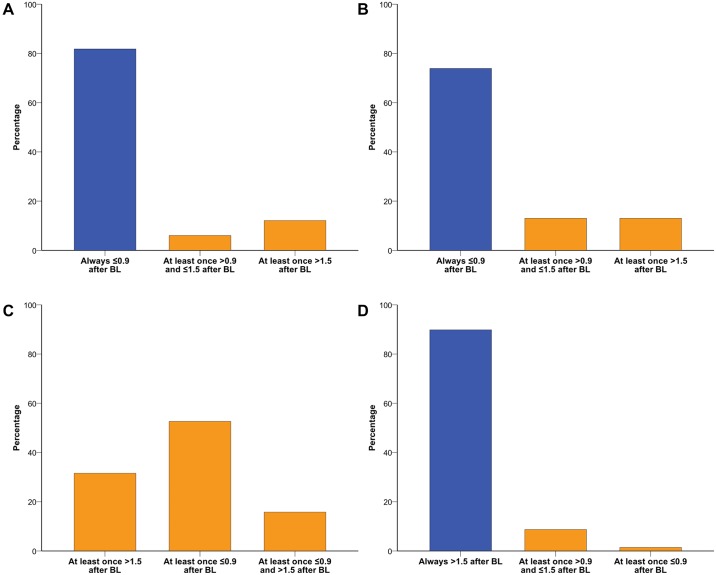
Percentages of patients switching between different anti-JCV antibody index categories. (A) Percentage of patients with anti-JCV antibody index ≤0.9 at baseline (n = 66) switching to higher index categories at least once during follow-up. (B) Percentage of patients with positive anti-JCV serostatus and antibody index ≤0.9 at baseline (n = 23) switching to higher index categories at least once during follow-up. (C) Percentage of patients with anti-JCV antibody index >0.9 and ≤1.5 at baseline (n = 19) switching to higher or lower index categories at least once during follow-up. There was no patient remaining within the baseline index category during follow-up. (D) Percentage of patients with anti-JCV antibody index >1.5 at baseline (n = 69) switching to lower index categories at least once during follow-up.

At baseline, 43 (27.9%) of 154 patients were anti-JCV antibody negative. Twelve (27.9%) of these 43 patients seroconverted from negative to positive anti-JCV antibody status at least once during follow-up; seven out of 12 (58.3%) patients reverted again to negativity at least once (Fig 3). Six (50%) of the 12 seroconverters remained consistently below anti-JCV antibody index level of ≤0.9, and 7 (58.3%) patients remained ≤1.5. Thus, 37 (86.0%) and 38 (88.4%) of the 43 patients with an anti-JCV antibody negative result at baseline remained either negative or consistently below the thresholds of ≤0.9 and ≤1.5, respectively, throughout follow-up. At least two consecutive samples with an anti-JCV antibody index >0.9 and >1.5 were observed only in 3 (7.0%) and 1 (2.3%) patient(s), respectively. Baseline anti-JCV antibody index was significantly lower in patients remaining seronegative at follow-up compared to those converting to seropositivity (median 0.16 vs. 0.24, p = 0.002, Fig 4A). ROC analysis revealed a high accuracy of baseline anti-JCV antibody index to predict stable negative serostatus (Fig 4A). Anti-JCV antibody indices in patients with baseline values ≤0.9 and ≤1.5, respectively, as well as predictive cut-off values according to a change to higher index category are shown in Fig 4C and 4D. Percentages of patients that switched from lower to higher anti-JCV antibody index categories are shown in Fig 5A–5C.

### Seroreversion

Of the 111 anti-JCV antibody positive patients at baseline, 14 (12.6%) seroreverted from positive to negative at least once; 13 (92.9%) of these patients converted again to positivity at least once (Fig 3). Baseline anti-JCV antibody index was higher in patients remaining seropositive at follow-up compared to those reverting to seronegativity (2.6 vs. 0.46, p<10^−7^; Fig 4B). Baseline anti-JCV antibody index showed a high accuracy to predict stable positive serostatus (Fig 4B). Percentages of patients that switched from higher to lower anti-JCV antibody index categories are shown in Fig 5C and 5D.

### Evolution of anti-JCV antibody index in natalizumab treated patients

A total of 31 patients received natalizumab therapy during the observation period, 29 started treatment after baseline visit. Accordingly, at baseline and prior to treatment initiation, 15 (51.7%) patients were anti-JCV antibody positive. Anti-JCV antibody index did not statistically significantly vary over time (median 65.9 months, IQR 60.2–70.8). Even though 8 patients (27.6%) changed initial serostatus at least once during follow-up, only 3 (10.3%) maintained the change (all seroconverters). Baseline anti-JCV antibody index was lower in patients remaining seronegative at follow-up compared to those converting to seropositivity (median 0.16 vs. 0.21, p = 0.07). Conversely, baseline anti-JCV antibody index was higher in patients remaining seropositive at follow-up compared to those reverting to seronegativity (2.4 vs. 0.3, p = 0.027).

The whole cohort of 154 patients was classified in patients with stable positive serostatus, with stable negative serostatus, with seroconversion or seroreversion after baseline. Multinomial logistic regression using age, sex, natalizumab treatment, and baseline anti-JCV antibody index as predictors provided evidence that the anti-JCV antibody index (p<10^−21^) and age (p<0.014) were significant predictors for change of anti-JCV serostatus. Regarding sex (p = 0.510) and treatment with natalizumab (p = 0.190) no statistically significant associations were found. Using the model the risk ratio of correctly predicted stable negative patients was 4.5 and of seroconverting patients 3.2 (Table 2).

**Table 2:** Power to predict stability or change of long-term anti-JCV antibody status.

Observed cases	Predicted cases				
	Stable negative	Converted to positivity	Reverted to negativity	Stable positive	Percent Correct
**Stable negative**	28	2	1	0	90.3%
**Converted to positivity**	8	3	1	0	25.0%
**Reverted to negativity**	1	1	7	5	50.0%
**Stable positive**	0	0	3	94	96.9%
**Overall Percentage**	24.0%	3.9%	7.8%	64.3%	85.7%

The observed and predicted cases in the respective patient groups are displayed. In the last column the percentage of correctly predicted cases by the multinomial logistic regression analysis is shown per patient group.

## Discussion

In this study, we extend previous data pertaining to the longitudinal development of anti-JCV antibody index in MS patients. We observed that overall anti-JCV antibody index remained stable over time, even though annual change of anti-JCV antibody status occurred in ~3% of patients. Furthermore, anti-JCV antibody index at baseline reliably predicted stable negative as well as stable positive JCV serostatus over a median follow-up of 64 months.

To date, several studies have reported different rates of seroconversion ranging from 2–15% per year [5, 8–14]. However, observation periods were usually short or only small numbers of longitudinal samples were available per patient; furthermore, seroreversion rates were not always described. In the present study a median of eleven longitudinally collected samples were available per patient over a median observation period of more than five years. Besides the duration of follow-up and frequency of sampling, the size of the cohort is one of the strengths of the present study. We found an annual rate of seroconversion of ~5.5% and seroreversion of ~2.5%. The fact that the rate of seroconversion is higher than of seroreversion matches the known observation that anti-JCV antibody prevalence [4–7, 10, 11] as well as anti-JCV antibody index values increase with patients’ age. The reasons why an increase of median anti-JCV antibody index over time could not be observed within the observation period are probably the overall wide spread of anti-JCV index values, the relatively small number of seroconverting patients, who showed mostly low anti-JCV antibody indices, and the time frame of five years that is probably too short to detect this effect—in contrast to patients’ age range.

There is a high precision of the anti-JCV antibody assay [20]. Previous studies have speculated that change of JCV serostatus, especially seroreversion, is at least partially due to the natural fluctuation of antibody levels among individuals near the cut-off point of the assay [5, 9, 11]. We provide further evidence for this hypothesis as 93% of seroreverters converted back again to positivity. These patients showed relatively low anti-JCV antibody indices at baseline (all but one patients had index values ≤0.9). Anti-JCV antibody negative patients, who seroconverted and later reverted again, showed slightly higher baseline anti-JCV antibody indices than patients remaining positive after initial seroconversion (data not shown); i.e. these patients were closer to the cut-off-point of the assay. Based on these findings, one might hypothesize that seroreversion does actually not exist (implying persistent anti-JCV immunoreactivity) and that true seroconversion is lower than currently assumed.

Previously, different anti-JCV antibody index categories have been defined for PML risk stratification and the cut points 0.9 and 1.5 included in the label of natalizumab [8, 21]–in the following termed as low (≤0.9), medium (>0.9 and ≤1.5) and high (>1.5) JCV index category. Due to this important clinical application, we have also used these categories and investigated the percentage of patients that switched between these different index categories. The majority of patients, who were anti-JCV antibody positive at baseline, remained within the same anti-JCV antibody index category throughout follow-up. For switching patients, it is difficult to draw reliable conclusions due to the small number per group. However, it seems that rather a similar percentage of patients with initial low index values switched to the medium and high group, while only few patients left the high index group to the medium group. The highest dynamics were found within the initial medium group.

In the present study, we could not confirm previous reports on higher seroconversion rates and rising anti-JCV antibody indices in MS patients treated with natalizumab [12, 14, 22]. Although we observed a numerically higher rate of serostatus change in natalizumab-treated patients compared to patients receiving other or no DMT, this difference was not statistically significant. The annual seroconversion rate of ~4% was lower than previously reported, and anti-JCV antibody index did also not increase over time in the natalizumab-treated cohort. Furthermore, the result of the multinomial logistic regression analysis underlines that not natalizumab treatment but anti-JCV antibody index and age determine change of future anti-JCV serostatus.

In practical terms, the false-negative rate, albeit small, in the assay combined with the possibility of seroconversion argues for the need of repetitive determination of anti-JCV serological status and antibody index in natalizumab-treated MS patients with an initial negative JCV serostatus. However, a patient tested once positive should be treated as always positive, as it is not attributable to either the insufficient discrimination power of the assay or a true anti-JCV immunoreactivity. When a patient switches between different JCV index categories, the highest ever-reached index category should be used to determine the individual PML risk.

There are some limitations of the study such as the retrospective design and selection of patients, which was based on availability of serum samples over at least four years follow-up. This could explain, why anti-JCV antibody prevalence was higher than previously reported. However, we did not intend to study anti-JCV antibody frequency; and different frequencies most likely do not impact on longitudinal evolution of antibody indices neither in the initially seronegative nor seropositive group. It cannot be excluded that the lower rate of seroconversion in the natalizumab-treated cohort (compared to other recent reports [12, 14, 22]) is due to the low number of patients in this treatment group.

This study provides evidence on the overall stability of anti-JCV antibody index and that anti-JCV antibody index tested once at baseline has a high predictive value for long-term stability of JCV serostatus. This allows the conclusion that already a singular anti-JCV antibody index can contribute to a reliable PML risk stratification and, thus, to individual benefit-risk evaluations for natalizumab-treated MS patients.

## References

[1] Polman CH, O'Connor PW, Havrdova E, et al A randomized, placebo-controlled trial of natalizumab for relapsing multiple sclerosis. *N Engl J Med*. 2006 354: 899–910. 10.1056/NEJMoa044397 16510744

[2] Tan CS, Koralnik IJ. Progressive multifocal leukoencephalopathy and other disorders caused by JC virus: clinical features and pathogenesis. *Lancet Neurol*. 2010 9: 425–437. 10.1016/S1474-4422(10)70040-5 20298966 PMC2880524

[3] Bloomgren G, Richman S, Hotermans C, et al Risk of natalizumab-associated progressive multifocal leukoencephalopathy. *N Engl J Med*. 2012 366: 1870–1880. 10.1056/NEJMoa1107829 22591293

[4] Olsson T, Achiron A, Alfredsson L, et al Anti-JC virus antibody prevalence in a multinational multiple sclerosis cohort. *Mult Scler*. 2013 19: 1533–1538. 10.1177/1352458513477925 23459571

[5] Trampe AK, Hemmelmann C, Stroet A, et al Anti-JC virus antibodies in a large German natalizumab-treated multiple sclerosis cohort. *Neurology*. 2012 78: 1736–1742. 10.1212/WNL.0b013e3182583022 22592369

[6] Bozic C, Richman S, Plavina T, et al Anti-John Cunnigham virus antibody prevalence in multiple sclerosis patients: baseline results of STRATIFY-1. *Ann Neurol*. 2011 70: 742–750. 10.1002/ana.22606 22162056

[7] Outteryck O, Zephir H, Salleron J, et al JC-virus seroconversion in multiple sclerosis patients receiving natalizumab. *Mult Scler*. 2013. 10.1177/1352458513505353 24072722

[8] Plavina T, Subramanyam M, Bloomgren G, et al Anti-JC virus antibody levels in serum or plasma further define risk of natalizumab-associated progressive multifocal leukoencephalopathy. *Ann Neurol*. 2014 76: 802–812. 10.1002/ana.24286 25273271 PMC4282070

[9] Gorelik L, Lerner M, Bixler S, et al Anti-JC virus antibodies: implications for PML risk stratification. *Ann Neurol*. 2010 68: 295–303. 10.1002/ana.22128 20737510

[10] Alroughani R, Akhtar S, Ahmed SF, et al JC virus seroprevalence and seroconversion in multiple sclerosis cohort: A Middle-Eastern study. *J Neurol Sci*. 2016 360: 61–65. 10.1016/j.jns.2015.11.044 26723975

[11] Aladro Y, Terrero R, Cerezo M, et al Anti-JC virus seroprevalence in a Spanish multiple sclerosis cohort: JC virus seroprevalence in Spain. *J Neurol Sci*. 2016 365: 16–21. 10.1016/j.jns.2016.03.050 27206867

[12] Vennegoor A, van Rossum JA, Leurs C, et al High cumulative JC virus seroconversion rate during long-term use of natalizumab. *Eur J Neurol*. 2016 23: 1079–1085. 10.1111/ene.12988 27018481

[13] Kolasa M, Hagman S, Verkkoniemi-Ahola A, Airas L, Koivisto K, Elovaara I. Anti-JC virus seroprevalence in a Finnish MS cohort. *Acta Neurol Scand*. 2016 133: 391–397. 10.1111/ane.12475 26347001

[14] Schwab N, Schneider-Hohendorf T, Pignolet B, et al Therapy with natalizumab is associated with high JCV seroconversion and rising JCV index values. *Neurol Neuroimmunol Neuroinflamm*. 2016 3: e195 10.1212/NXI.0000000000000195 26848486 PMC4733149

[15] Polman CH, Reingold SC, Banwell B, et al Diagnostic criteria for multiple sclerosis: 2010 revisions to the McDonald criteria. *Ann Neurol*. 2011 69: 292–302. 10.1002/ana.22366 21387374 PMC3084507

[16] Miller DH, Weinshenker BG, Filippi M, et al Differential diagnosis of suspected multiple sclerosis: a consensus approach. *Mult Scler*. 2008 14: 1157–1174. 10.1177/1352458508096878 18805839 PMC2850590

[17] Lublin FD, Reingold SC. Defining the clinical course of multiple sclerosis: results of an international survey. National Multiple Sclerosis Society (USA) Advisory Committee on Clinical Trials of New Agents in Multiple Sclerosis. *Neurology*. 1996 46: 907–911. 8780061 10.1212/wnl.46.4.907

[18] Kister I, Kuesters G, Chamot E, et al IV immunoglobulin confounds JC virus antibody serostatus determination. *Neurol Neuroimmunol Neuroinflamm*. 2014 1: e29 10.1212/NXI.0000000000000029 25340081 PMC4204227

[19] Lee P, Plavina T, Castro A, et al A second-generation ELISA (STRATIFY JCV DxSelect) for detection of JC virus antibodies in human serum and plasma to support progressive multifocal leukoencephalopathy risk stratification. *J Clin Virol*. 2013 57: 141–146. 10.1016/j.jcv.2013.02.002 23465394

[20] Plavina T, Berman M, Njenga M, et al Multi-site analytical validation of an assay to detect anti-JCV antibodies in human serum and plasma. *J Clin Virol*. 2012 53: 65–71. 10.1016/j.jcv.2011.10.003 22104399

[21] Tysabri. Product information. http://wwwemaeuropaeu/docs/en_GB/document_library/EPAR_-_Product_Information/human/000603/WC500044686pdf. Accessed 23 Jan 2017.

[22] Raffel J, Gafson AR, Malik O, Nicholas R. Anti-JC virus antibody titres increase over time with natalizumab treatment. *Mult Scler*. 2015 21: 1833–1838. 10.1177/1352458515599681 26449743

